# CARING FOR CANCER PATIENTS LIVING WITH DEMENTIA: CHALLENGES AND WAYS FORWARD

**DOI:** 10.1093/geroni/igae098.3069

**Published:** 2024-12-31

**Authors:** Shelley Canning, Michael McKenzie, Genevieve St-Martin, Jagbir Kaur, Nicole Percival, Lillian Hung, Lynn Jackson, Rachel Wan

**Affiliations:** University of the Fraser Valley, Abbotsford, British Columbia, Canada; BC Cancer, Vancouver, British Columbia, Canada; BC Cancer, Abbotsford, British Columbia, Canada; BC Cancer, Vancouver, British Columbia, Canada; Maplewood Care Society, Abbotsford, British Columbia, Canada; University of British Columbia, Vancouver, British Columbia, Canada; University of British Columbia, Vancouver, British Columbia, Canada; Vancouver Coastal Health, Vancouver, British Columbia, Canada

## Abstract

The purpose of this study is to address the inequity of care outcomes for patients living with both cancer and dementia through exploring current challenges and barriers, with the goals of creating dementia-friendly cancer care practice recommendations and education. Cancer patients also living with dementia have been found to experience poor outcomes, receiving less cancer screening, staging, and curative treatment than patients without dementia, thus they are typically diagnosed at later stage cancers with lower survival rates. We are conducting a qualitative applied health study drawing on Interpretive Description methodology underpinned by a person-centred philosophy. Phase one of the study has involved conducting interviews with patients, their care-givers, and cancer care providers. In this presentation we are focusing on the findings from our interviews with cancer care providers. Our sample includes 15 RNs, and 15 participants including physicians, therapists, and counsellors. Although they are experts in cancer, care providers often lack confidence and knowledge regarding dementia-care. Findings from their interviews led to themes reflecting both challenges and barriers to providing care. Specific themes related to care challenges include: Difficulty Following Instructions; Balancing Treatment Risks with QOL; Including Care-givers; and Ethical Dilemmas. Additionally, specific themes related to barriers include: Lack of Communication Processes; Lack of Continuity of Care; Lack of Resources; and Lack of Dementia Education. These themes will inform Phase two of the study which will involve creating dementia aware education and recommendations for dementia-friendly policies and processes to better tailor cancer care for patients living with dementia ensuring equity.

